# Bifascicular block in unexplained syncope is underrecognized and under-evaluated: A single-center audit of ESC guidelines adherence

**DOI:** 10.1371/journal.pone.0263727

**Published:** 2022-02-28

**Authors:** Muhammad Asim Shabbir, Muhammad Hamza Saad Shaukat, Moiz Ehtesham, Shannon Murawski, Sukhraj Singh, Rizwan Alimohammad

**Affiliations:** 1 Department of Internal Medicine, Albany Medical College, Albany, NY, United States of America; 2 Department of Cardiology, Capital Cardiology Associates, Albany Medical College, Albany, NY, United States of America; Johns Hopkins University School of Medicine, UNITED STATES

## Abstract

**Background:**

The presence of bifascicular block on electrocardiography suggests that otherwise-unexplained syncope may be due to complete heart block. European Society of Cardiology (ESC) recommends investigating it with electrophysiology study (EPS). PPM is indicated if high-degree atrioventricular block is inducible. Long term rhythm monitoring with implantable loop recorder (ILR) is recommended if EPS is negative. We evaluated adherence to these guidelines.

**Methods:**

This is a single-center retrospective audit of adult patients with bifascicular block hospitalized for unexplained syncope between January 2018 and August 2019 under general medicine service. Patients with an alternative explanation for syncope were excluded. Guideline adherence was assessed by formal cardiology consult and whether EPS followed by ILR and/or PPM were offered.

**Results:**

65 out of 580 adult patients (11.2%) admitted to general medicine service for syncope had a bifascicular block; 29 (5%) were identified to have bifascicular block and unexplained syncope. Median age was 77 ±10 years; 9 (31%) were female, and 6 (20.7%) patients had at least one prior hospital visit for syncope at our academic medical center. Cardiology was consulted on 17 (58.6%) patients. Two patients were evaluated by EPS (1 refused) followed by ILR. Overall, 3 out of 29 patients (10.3%) received guideline-directed evaluation during the hospitalization based on ESC guidelines. None of the patients received empiric PPM during the index hospitalization.

**Conclusion:**

Among patients admitted to the general medicine service with unexplained syncope and bifascicular block, a minority (10.3%) underwent guideline-directed evaluation per ESC recommendations. Cardiology was consulted in 58.6% of cases.

## Introduction

**Fig 1:**
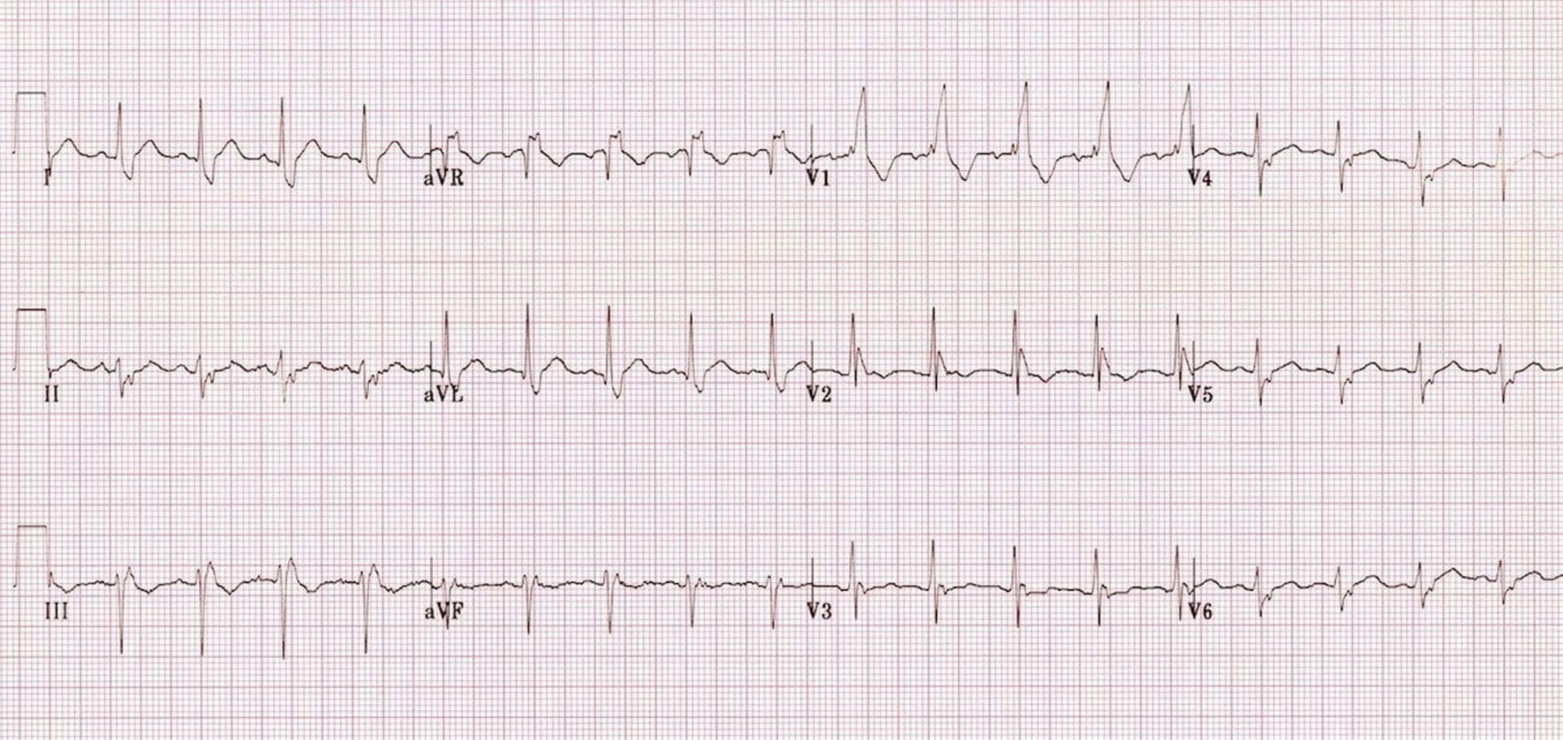
ECG showing bifascicular block (left anterior fascicular block and right bundle branch block).

Bifascicular block is defined on electrocardiography as left bundle branch block, right-bundle branch block with left anterior fascicular block (Fig 1), or right bundle branch block with left posterior fascicular block. The presence of bifascicular block on electrocardiography suggests that otherwise-unexplained syncope may be due to complete heart block. However, the incidence of high-degree atrioventricular block in patients with bifascicular block is unclear and estimated to be less than 50% [1]. The European Society of Cardiology (ESC) recommends investigating it with electrophysiology study (EPS) [2]. Permanent pacemaker (PPM) is indicated if baseline HV interval ≥ 70ms or high-degree atrioventricular block is induced by incremental atrial pacing or pharmacologic stress. Long-term rhythm monitoring with implantable loop recorder (ILR) is recommended if EPS is negative.

We conducted an audit at our university-based academic tertiary-care hospital to assess adherence to ESC guidelines for adult patients with bifascicular block hospitalized admitted under Internal Medicine service for unexplained syncope.

## Methods

This is a single-center retrospective audit. Electronic medical records of adult patients admitted and discharged by general medicine service with a primary diagnosis of syncope between January 2018 and August 2019 were reviewed. Patients with chronic bifascicular block (confirmed on at least one prior electrocardiogram) and unexplained syncope were identified after thorough chart review including history, physical exam, lab data, electrocardiogram (EKG), echocardiography, and discharge summary. Exclusion criteria were: pre-existing pacemaker; supraventricular or ventricular arrhythmia, or second/third-degree atrioventricular block, bradycardia (heart rate <50 beats per min) with or without the use of negative chronotropic medications; left ventricular ejection fraction < 35%, orthostatic hypotension, vasovagal syncope per history, seizure or recent cerebrovascular accident, cardiac ischemia or infarction related syncope; hypertrophic, infiltrative or inflammatory cardiomyopathy; moderate to severe valvular disease (primary or secondary); abnormal serum magnesium or potassium levels at presentation (Table 1)–patients fulfilling at least 1 criterion were excluded since there may be an explanation for syncope other than high-degree atrioventricular block owing to bifascicular block. Patients who had unexplained syncope on admission but had later identified to have an alternative cause of syncope during the hospital course were also excluded.

**Table 1:** Pre-specified exclusion criteria for diagnosis of unexplained syncope.

Pre-existing pacemaker
Documented arrhythmia^a^, or second/third-degree atrioventricular block
Bradycardia (heart rate <50 beats per min) with or without the use of negative chronotropic medications
Left ventricular ejection fraction < 35%
Orthostatic hypotension, vasovagal syncope per history, seizure or recent cerebrovascular accident, cardiac ischemia or infarction related syncope
Hypertrophic, infiltrative or inflammatory cardiomyopathy
Moderate to severe valvular disease (primary or secondary)
Abnormal serum magnesium or potassium levels at presentation

^a^supraventricular or ventricular.

Guideline adherence was assessed by formal cardiology consult and whether EPS followed by PPM or ILR were offered. Chi-square test of independence and Fischer’s exact test were performed for statistical analysis.

The audit was approved by the institutional review board. There was no patient and public involvement in the design, or conduct, or reporting, or dissemination plans of the research.

## Results

65 out of 580 consecutive adult patients (11.2%) admitted to general medicine service for syncope had a bifascicular block confirmed on EKG; 29 (5%) were identified to have bifascicular block with no alternative explanation of syncope (unexplained syncope). Fifteen patients (51.7%) had a left bundle branch block. The median age was 77±10 years; 9 (31%) were female, and 6 (20.7%) patients had at least one prior hospital visit for syncope at our academic medical center. The baseline characteristics for the 29 patients with unexplained syncope and bifascicular block are summarized in Table 2.

**Table 2:** Baseline characteristics of the study population.

Baseline characteristics	Patients with guideline-directed evaluation^*a*^ (n = 3)	Patients without guideline-directed evaluation (n = 26)	p-value
**Age (Median ± standard deviation) in years**	79 ± 5	74±11	0.48
**Female**	1 (33.3%)	8 (30.8%)	0.92
**Type of bifascicular block:**			
• **Left bundle branch block**	1 (33.3%)	14 (53.8%)	0.50
• **Right bundle branch block, & left anterior or posterior fascicular block**	2 (66.7%)	12 (46.2%)	0.50
**≥1 prior unexplained syncope related hospitalization within last 12 months**	0 (0%)	6 (23.1%)	1.0
**Atrial fibrillation**	2 (66.7%)	8 (30.8%)	0.21
**Hypertension**	2 (66.7%)	22 (84.6%)	0.43
**Diabetes Mellitus**	1 (33.3%)	7 (27%)	0.81
**Dyslipidemia**	3 (100%)	22 (84.6%)	1.0
**Current or former smoker**	1 (33.3%)	18 (69.2%)	0.21
**Chronic kidney disease (GFR<60mlmin/1.75m** ^**2**^ **)**	2 (66.7%)	7 (27%)	0.15
**Known coronary artery disease**	1 (33.3%)	10 (34.5%)	0.86

^a^Electrophysiologic study followed by long-term cardiac monitor or pacemaker.

**Fig 2:**
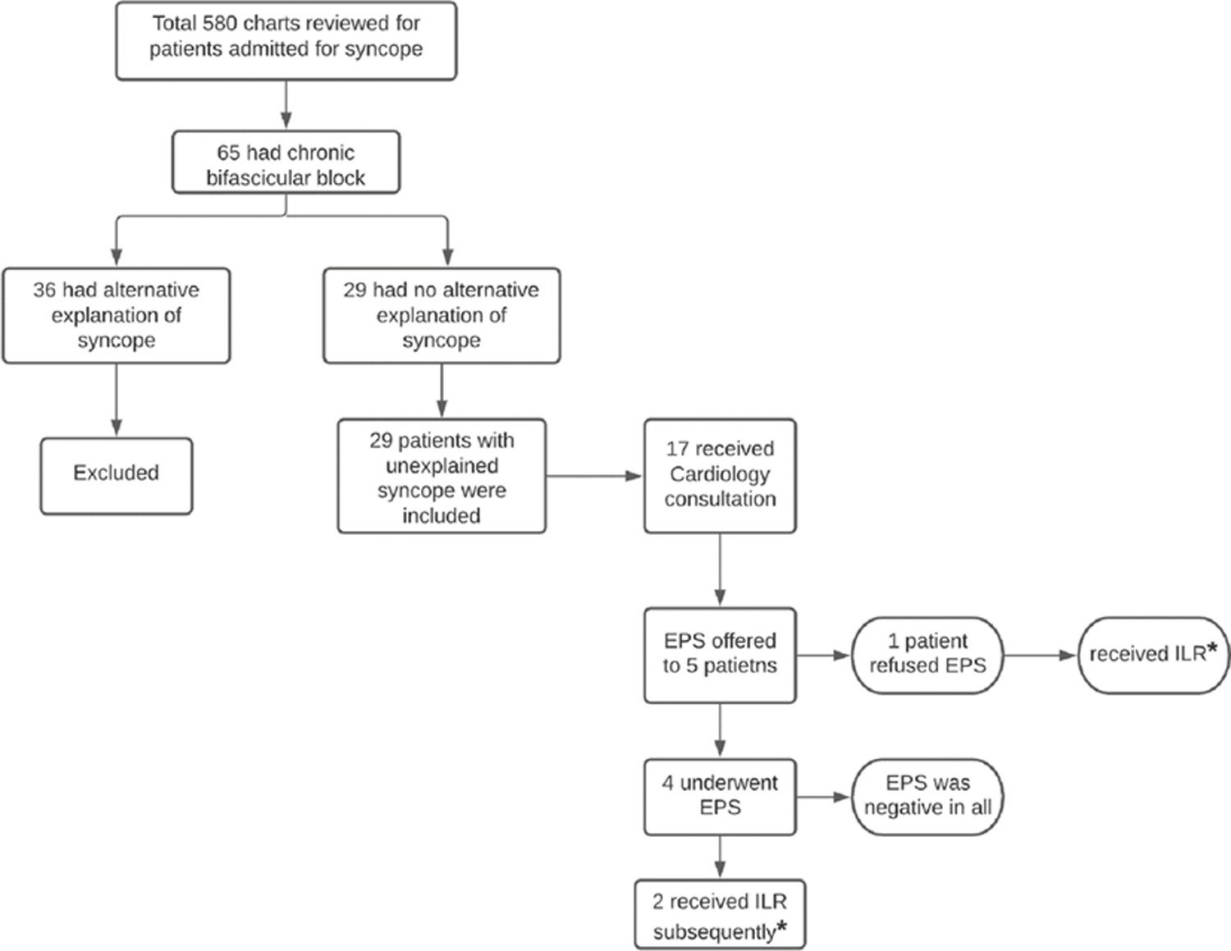
The summary of study cohort with respect to ESC guideline-directed evaluation. *Patients evaluated appropriately per ESC guideline-directed evaluation.

Cardiology was consulted on 17 (58.6%) patients. EPS was offered to 5 patients; 1 refused and opted for ILR directly. EPS was negative in all four patients who underwent testing; 2/4 patients were subsequently offered (and received) ILR and the rest were not offered further treatment. Of the total cohort, 5 patients were directly offered ILR without EPS. Overall, 3 (*1 refusing EPS and opting for ILR + 2 with negative EPS subsequently offered ILR*) out of 29 patients (10.3%) with bifascicular block and unexplained syncope received guideline-directed evaluation during the hospitalization based on ESC guidelines (Fig 2). All patients evaluated appropriately received cardiology consultation, 100% vs. 53.8% (p = 0.24). Overall, the diagnosis of bifascicular block (or left bundle branch block) was documented on the discharge summary of 12 out of 29 patients with unexplained syncope (41.4%).

## Discussion

With an increasing focus on preventing inappropriate hospitalizations, syncope is the leading diagnosis associated with Medicare and Medicaid payment denials in the US [3–7]. Bifascicular block is recognized as a high-risk electrocardiographic feature for cardiac syncope in both ESC and American College of Cardiology/Heart Rhythm Society (ACC/HRS) guidelines [2, 8]. There are some differences in the European and US recommendations for the evaluation of bifascicular block in unexplained syncope, the latter favoring empiric pacemaker use [8, 9]. On retrospective review of unexplained syncope in patients with bifascicular block admitted to general medicine service over 20 months (January 2018 –August 2019), 10.3% underwent ESC guideline-directed evaluation. None of the patients received empiric PPM during the index hospitalization (ACC/HRS recommendation). In this audit, only 41.4% of patients with bifascicular block and unexplained syncope had documentation of the diagnosis of bifascicular block or left bundle branch block on discharge summary highlighting the need for increased awareness. This may explain less frequent cardiology consultation. Cardiology consultation increased the likelihood of appropriate evaluation compared to the patients not seen by cardiology team by 17.6% (3/17) vs.0% (0/12), p = 0.274. The lack of understanding of diagnosing bifascicular block may contribute to the low frequency of cardiology consultations. Automatic EKG readings can be misleading, and the diagnosis of bifascicular block may not always be correctly labeled. Documenting bifascicular block as a risk factor for cardiac syncope may help insurance claims for payment in patients without traditional risk factors.

While the prevalence of bifascicular block with unexplained syncope among all syncope admissions (5% in this audit) remains unclear and previously unreported, the incidence of unexplained syncope in patients with bifascicular block is estimated to be 5–8% [10, 11]. For general medicine physicians (internists) admitting patients for syncope, recognition, and appropriate evaluation of bifascicular block in unexplained syncope has the dual benefit of justifying hospitalizations for suspected cardiac syncope in patients without traditional cardiac risk factors and preventing recurrent syncope-related trauma/hospitalization.

Our audit helps demonstrate the value of appropriate evaluation with EPS or ILR–currently being studied in the SPRITELY trial, [12] and highlights the need for awareness of guidelines amongst internists. Since there were no patients who received empiric PPM based on ACC/HRS guidelines, its benefit cannot be inferred from this study. We did not find published reports of guideline adherence patterns, in the US or elsewhere, for evaluation of bifascicular block in unexplained syncope. The limitations of our audit include a retrospectively analysis, limited generalization of the findings given single center, and small sample size. Bifascicular block and unexplained syncope, as referenced above, are rare. Studies that have evaluated the role of pacing and/or loop recorder in bifascicular block enrolled around 100 patients–remarkably small numbers for international, multi-center prospective trials [12, 13].

## Supporting information

S1 Data (XLSX)

## References

[1] Brignole M., et al., Mechanism of syncope in patients with bundle branch block and negative electrophysiological test. Circulation, 2001. 104(17): p. 2045–50. doi: 10.1161/hc4201.097837 11673344

[2] Brignole M., et al., 2018 ESC Guidelines for the diagnosis and management of syncope. European Heart Journal, 2018. 39(21): p. 1883–1948. doi: 10.1093/eurheartj/ehy037 29562304

[3] RACTrac survey: Exploring the impact of the RAC program on hospitals nationwide | AHA. (n.d.). Retrieved from https://www.aha.org/guidesreports/2012-02-15-ractrac-survey-exploring-impact-rac-program-hospitals-nationwide

[4] Sun B.C., et al., Randomized clinical trial of an emergency department observation syncope protocol versus routine inpatient admission. Ann Emerg Med, 2014. 64(2): p. 167–75. doi: 10.1016/j.annemergmed.2013.10.029 24239341 PMC4019722

[5] Angus S., The Cost-Effective Evaluation of Syncope. Med Clin North Am, 2016. 100(5): p. 1019–32. doi: 10.1016/j.mcna.2016.04.010 27542422

[6] Saad Shaukat M.H., et al., Is our initial evaluation of patients admitted for syncope guideline-directed and cost-effective? European heart journal. Case reports, 2020. 4(2): p. 1–4. 10.1093/ehjcr/ytaa032 PMC7180576 32352069

[7] Sun B.C., Emond J.A., and Camargo C.A. Jr., Direct medical costs of syncope-related hospitalizations in the United States. Am J Cardiol, 2005. 95(5): p. 668–71. doi: 10.1016/j.amjcard.2004.11.013 15721118

[8] Shen W.K., et al., 2017 ACC/AHA/HRS Guideline for the Evaluation and Management of Patients With Syncope: A Report of the American College of Cardiology/American Heart Association Task Force on Clinical Practice Guidelines and the Heart Rhythm Society. Circulation, 2017. 136(5): p. e60–e122. doi: 10.1161/CIR.0000000000000499 28280231

[9] Goldberger ZD, Petek BJ, Brignole M, et al. Acc/aha/hrs versus esc guidelines for the diagnosis and management of syncope: jacc guideline comparison. J Am Coll Cardiol. 2019;74(19):2410–2423. doi: 10.1016/j.jacc.2019.09.012 31699282

[10] Dhingra R.C., et al., Syncope in patients with chronic bifascicular block. Significance, causative mechanisms, and clinical implications. Ann Intern Med, 1974. 81(3): p. 302–6. doi: 10.7326/0003-4819-81-3-302 4854561

[11] McAnulty J.H., et al., Natural history of "high-risk" bundle-branch block: final report of a prospective study. N Engl J Med, 1982. 307(3): p. 137–43. doi: 10.1056/NEJM198207153070301 7088050

[12] Krahn A.D., et al., Empiric pacemaker compared with a monitoring strategy in patients with syncope and bifascicular conduction block—rationale and design of the Syncope: Pacing or Recording in ThE Later Years (SPRITELY) study. Europace, 2012. 14(7): p. 1044–8. doi: 10.1093/europace/eus005 22318881

[13] Santini M., et al., Prevention of Syncope Through Permanent Cardiac Pacing in Patients With Bifascicular Block and Syncope of Unexplained Origin. Circulation: Arrhythmia and Electrophysiology, 2013. 6(1): p. 101–107. doi: 10.1161/CIRCEP.112.975102 23390123

